# The Necessity of a State Pediatric Society

**Published:** 1899-07

**Authors:** Oliver B. Bush

**Affiliations:** Colquitt, Ga.


### THE NECESSITY OF A STATE PEDIATRIC SOCIETY.*

BY OLIVER B. BUSH, M.D.,

COLQUITT, GA.

The subject of which I am about to speak is one of the utmost importance and one which has been but little agitated in this part of the United States among the medical profession, and the object of this paper is to impress upon the profession the great importance of studying and treating the diseases of children above all others. It is of great importance for several reasons. 1st. Because the class of individuals referred to are so important, not only because they are human beings and sufferers in common with all mankind, but far more so because they are the most helpless; and still more so because they are the angels of the fireside and the hope of the country.

Am I exaggerating when I call them the angels of the fireside? No; because the Saviour said, "of such is the kingdom of heaven."

Mr. President, after the labors of the day have been ended and we wend our way homeward at night, the babe with its outstretched arms and lovely prattle is the first of the family to engage our attention, and while in health is the delight of all; but, on the contrary, when we walk in and find the little darling sick, imagine the agony and suspense of that father who loves the

*Read before the Georgia Medical Association at Macon, Ga., on April 19-21.

little fellow so dearly; why, there is no sacrifice he would not make for the relief of that babe.

The doctor, of course, is the first person thought of after the good motherly neighbors have been consulted, and now imagine the suspense of the poor doctor when he is called in and the patient unable to give him a single symptom. Of course he appeals to the mother who, in a disconnected and unsatisfactory manner, proceeds (as best she can) to give the history of the case.

The diseases of childhood are as various as those of the adult and at all times more difficult of diagnosis; especially is this so with the young practitioner, and it is not infrequently the case that he (especially in the remote parts of the State) would like to read and have discussed before the association a paper bearing on some disease of childhood; yet he does not do so because he fears there would be so little interest taken in this class of troubles that he would hardly have time to read the paper, much less have discussions on it.

According to the by-laws of this association we only have three days in which to discuss the various subjects—relating mostly to adults—and having reports of committees, etc., etc.; and there is not time enough to devote to the discussion of diseases of children. Therefore, Mr. President, is it not advisable that a society for the consideration of this class of diseases alone be organized, or if this is not practicable, to extend the time of this association and a special section be inaugurated to consider this class of diseases? Now when we take into consideration the vast number of diseases that the child is heir to, and the limited time we have in this association to discuss them in and learn each others' views relative to their treatment, we are more impressed with the great importance of a society for the consideration of this special class of practice.

There is not a practitioner in this whole country who will not testify with me that our facilities for studying the diseases of children in our colleges are too meager and that our chances to become familiar with them are only from bedside observation and based almost alone upon our individual experience, and how often is it the case that we have even doubted our own diagnosis?

Imagine the great remorse of the sympathetic practitioner when he sees his own mistake and when he feels that many lives have been sacrificed in an ignorant and experimental way.

To lengthen the time of meeting of our Medical Association will require the alteration of the Constitution of said association, and I think that this would be inappropriate because of the value of the time of a large number of the members and the necessity for them to be at home.

After a careful perusal of the statistics our information is that we only have two State pediatric societies in the United States, viz.: one in Ohio and one in Indiana, and so far as our information extends at present, there are only two cities which have organized pediatric societies, viz.: The Bethesda of St. Louis, and the Philadelphia.

The interest taken in these pediatric studies is increasing, and really astonishing. Now in support of the claim for a state pediatric society in Georgia there are, according to our information, based on the last census, an average of two or three children to each family in Georgia, which will make the number of children run up into the hundreds of thousands; so you see the number of this class of patients far exceeds that of the adults, and as we are called upon from day to day to attend these little sufferers, and they oftentimes are so small as not to be able to tell us anything of their condition or give us a history of their case; then of course we have to judge from symptoms alone as to their troubles. Therefore it becomes necessary to watch the symptoms as they arise, and base our diagnosis thereon as we do the spoken symptoms of the adult.

In the study of the diseases of childhood as well as the treatment of the same, supposition must necessarily enter largely into the case. I say supposition, for it is, as we have no way of telling positively the trouble otherwise. In view of the foregoing facts and for the better preparing ourselves for the treatment of the diseases of children, I suggest that we need a state pediatric society, with its rules, regulations and by-laws to govern the same, as other associations have, in which no diseases except those peculiar to childhood should be discussed.

We notice again after a further perusal of the statistics that there are, out of one hundred and thirty (only thirteen not being heard from), one hundred and seventeen regular medical colleges in the United States. There is only about 54 or 55 per cent. of these which have a chair of pediatrics for the special study of diseases of children. There is about 36 or 37 per cent. which have the chair of pediatrics combined with some other branch of study. This last estimate of course does not include the study of diseases of children as the minor subject. We understand also that there is only a lectureship on pediatrics of about three or four in the United States.

There is no announced method of teaching of pediatrics in about ten of these colleges. However, we are of the opinion that in these ten the study of the diseases of children is required. So we therefore see that while the different colleges recognize the importance of teaching this branch of study, the facilities are very poor and altogether inadequate for doing so. We notice in the schools of even our individual State that we have the clinical profess·r or professor of clinical diseases of children or lecturer on the diseases of children, but where and in what manner is this taught? In most colleges it is only an adjunct to the chair of practice. This goes to show within itself the recognition of the necessity of the study of the diseases of children and also the importance of bedside observations.

I think that the diseases of children should not only be taught didactically, but clinically.

I think not only should pediatrics be taught in the schools of our state, but it should be regarded as much of a necessity as the chair of practice or surgery, and it should be made a branch of the colleges which require a final examination by the professors of that chair.

There is no class of physicians who feel the need of such an organization so keenly as the country practitioner. We should study it, teach it, write about it, discuss it at our meetings, practice it as we country physicians have to do, that we may by so doing fit ourselves, and others, to aid the most helpless, the most defenseless, the most pitiful and the most appealing of all our patients.

As physicians we know that we cannot save the life of a child or adult but by the revealed experience of others and the close study of this the most important branch of medical science. We can cut short diseases that would if let alone produce death.

There is a large number of physicians of the country as well as in the cities who do not know what diseases they are treating when they are called to see a child, on account of the obscurity of the symptoms. The consequence is the mortality among these little sufferers is appalling sometimes.

Now in view of the above fact, would it be amiss to suggest the formation of a pediatric society, or, if this is not agreed to, that a section be added to this association especially for the reading of reports of cases and papers and for their discussion?

It seems to me to be imperative.

